# Beyond the Double-Strand Breaks: The Role of DNA Repair Proteins in Cancer Stem-Cell Regulation

**DOI:** 10.3390/cancers13194818

**Published:** 2021-09-26

**Authors:** Jacqueline Nathansen, Felix Meyer, Luise Müller, Marc Schmitz, Kerstin Borgmann, Anna Dubrovska

**Affiliations:** 1 OncoRay-National Center for Radiation Research in Oncology, Faculty of Medicine and University Hospital Carl Gustav Carus, Technische Universität Dresden and Helmholtz-Zentrum Dresden-Rossendorf, 01309 Dresden, Germany; 2 Laboratory of Radiobiology & Experimental Radiooncology, Department of Radiotherapy and Radiation Oncology, Center of Oncology, University Medical Center Hamburg-Eppendorf, 20246 Hamburg, Germany; fe.meyer@uke.de (F.M.); borgmann@uke.de (K.B.); 3 Institute of Immunology, Faculty of Medicine Carl Gustav Carus, TU Dresden, 01307 Dresden, Germany; luise.mueller1@tu-dresden.de (L.M.); marc.schmitz@tu-dresden.de (M.S.); 4 National Center for Tumor Diseases (NCT), Partner Site Dresden of the German Cancer Research Center (DKFZ), Faculty of Medicine and University Hospital Carl Gustav Carus, Technische Universität Dresden and Helmholtz Association/Helmholtz-Zentrum Dresden-Rossendorf (HZDR), 01307 Dresden, Germany; 5 German Cancer Consortium (DKTK), Partner Site Dresden, and German Cancer Research Center (DKFZ), 69120 Heidelberg, Germany; 6 Helmholtz-Zentrum Dresden-Rossendorf, Institute of Radiooncology-OncoRay, 01328 Dresden, Germany

**Keywords:** cancer stem cells, DNA repair, replicative stress, oxidative stress, genomic instability, immune response, reprogramming, cancer therapy

## Abstract

**Simple Summary:**

Cancer stem cells (CSCs) are a tumor cell population maintaining tumor growth and promoting tumor relapse if not wholly eradicated during treatment. CSCs are often equipped with molecular mechanisms making them resistant to conventional anti-cancer therapies whose curative potential depends on DNA damage-induced cell death. An elevated expression of some key DNA repair proteins is one of such defense mechanisms. However, new research reveals that the role of critical DNA repair proteins is extending far beyond the DNA repair mechanisms. This review discusses the diverse biological functions of DNA repair proteins in CSC maintenance and the adaptation to replication and oxidative stress, anti-cancer immune response, epigenetic reprogramming, and intracellular signaling mechanisms. It also provides an overview of their potential therapeutic targeting.

**Abstract:**

Cancer stem cells (CSCs) are pluripotent and highly tumorigenic cells that can re-populate a tumor and cause relapses even after initially successful therapy. As with tissue stem cells, CSCs possess enhanced DNA repair mechanisms. An active DNA damage response alleviates the increased oxidative and replicative stress and leads to therapy resistance. On the other hand, mutations in DNA repair genes cause genomic instability, therefore driving tumor evolution and developing highly aggressive CSC phenotypes. However, the role of DNA repair proteins in CSCs extends beyond the level of DNA damage. In recent years, more and more studies have reported the unexpected role of DNA repair proteins in the regulation of transcription, CSC signaling pathways, intracellular levels of reactive oxygen species (ROS), and epithelial–mesenchymal transition (EMT). Moreover, DNA damage signaling plays an essential role in the immune response towards tumor cells. Due to its high importance for the CSC phenotype and treatment resistance, the DNA damage response is a promising target for individualized therapies. Furthermore, understanding the dependence of CSC on DNA repair pathways can be therapeutically exploited to induce synthetic lethality and sensitize CSCs to anti-cancer therapies. This review discusses the different roles of DNA repair proteins in CSC maintenance and their potential as therapeutic targets.

## 1. Introduction

Recent discoveries for cancer therapy, such as antibody-based immunotherapy, and various predictive biomarkers, have shifted the focus from standard uniform treatment towards personalized approaches [1,2]. Although significant improvement in life expectancy has been reached for certain tumors, tumor relapses still represent a major threat for patients with metastatic disease [3,4,5]. Mounting evidence suggests that recurrences can be attributed, at least in part, to the existence of a pluripotent subpopulation of tumor cells. The cancer stem cells (CSCs) possess the ability to self-renew and exhibit an enhanced therapy resistance. Therefore, they can re-populate a tumor after initially successful therapy [5,6,7,8]. Since their first isolation from acute myeloid leukemia by the group of John Dick, CSCs have been identified in various solid tumors [9,10]. As with their non-malignant stem-cell counterparts, CSCs are equipped with various mechanisms to protect their genome from endogenous or treatment-induced damage [6,11].

Less DNA damage in CSCs as compared to their non-CSC counterparts was reported in several studies that quantified DNA damage after genotoxic therapies or at baseline levels. This quantification was made, e.g., using residual γH2A.X foci analysis (for example, more efficient foci resolution was shown for lung cancer CD133^+^ cells [12], murine cancer CD29^+^CD24^high^ cells [13], head and neck squamous cell carcinoma (HNSCC) ALDH^+^ cells [14], glioblastoma CSC populations in patient-derived cell lines [15]) or by comet assay (for example the lower amount of DNA damage was found in glioblastoma patient-derived CD133^+^ cells [16], CD133^+^CD44^+^ colon cancer cells [17] and murine cancer CD29^+^CD24^high^ cells [13]). However, some studies showed no difference in the levels of DNA damage quantified in the same way in CSCs and non-CSC populations [18,19,20]. This controversy can be partially attributed to DNA repair pathways that can be activated independently on H2AX phosphorylation [21], different timepoints used for these analyses, inconsistent methods for CSC isolation or enrichment, and, in some cases, lack of CSC functional validation. At the same time, quantification of DNA damage and DNA repair response does not always correlate with tumor resistance to the DNA-damaging treatment [19,20,22].

Interestingly, while the accumulation of mutations is a major threat for the maintenance of embryonic and adult stem-cell populations, a certain level of genomic instability can promote malignant transformation and CSC induction [23,24]. According to the unified model of tumor evolution suggested by Kreso and Dick, CSCs are located at the apex of the tumor cell hierarchy [8]. Yet, the ongoing process of tumor evolution enables CSCs to acquire additional mutations that further enhances their tumorigenic potential and therapy resistance [8]. Hereditary mutations in key DNA repair genes, such as the breast cancer susceptibility (BRCA) and Fanconi anemia (FA) genes, drastically emphasize the role of genomic instability as a driving force in tumorigenesis [23,25,26]. Importantly, previous reports suggest that this relationship is not linear. Long-term accumulation of DNA damage can lead to permanent replication stress and intolerable mutation burden, eventually activating tumor suppression mechanisms such as apoptosis and senescence [27,28,29]. In this context, suppression of the DNA damage response (DDR) and its associated signaling might increase the DNA damage tolerance and prolongs the survival of CSCs [20,22,30]. However, various studies demonstrated that CSCs crucially rely on the up-regulation of DNA repair pathways to counteract the adverse effects of genomic instability, as reviewed in detail elsewhere [6,11,31]. Consequently, the high basal levels of cell cycle checkpoint kinases and DNA repair proteins provide CSCs with a robust armor against genotoxic treatment. Yet, the dependency on a balanced DDR also provides promising opportunities for targeted therapy, as demonstrated for poly(ADP-ribose) polymerase 1 (PARP1) inhibitors in BRCA-mutant tumors [32].

**Figure 1:**
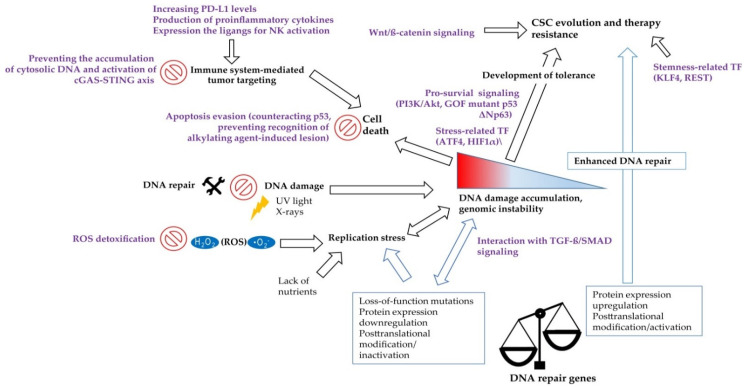
Canonical and non-canonical role of DNA repair proteins in the cancer stem-cell (CSC) phenotype. The dysregulated expression and activation of DNA repair proteins influence CSC evolution and therapy resistance via. their canonical function in the repair of exogenous and endogenous DNA damage and the prevention of replication stress (blue boxes/arrows). Their multiple non-DNA repair-related roles include interaction with CSC signaling and stemness-related transcription factors (TF), reactive oxygen species (ROS) detoxification and immune evasion (violet). Akt—Protein kinase B; ATF4—Stress-related activating transcription factor 4; cGAS—GMP—AMP synthase; GOF—Gain-of-function mutant; HIF1α—Hypoxia-inducible factor 1 alpha; KLF4—Krüppel-like factor 4; PI3K—Phosphatidylinositol-3-kinase; NK—Natural killer cells; PD-L1—Programmed death-ligand 1 (PD-L1); REST—Repressor element 1 silencing transcription factor; SMAD3—Mothers against decapentaplegic homolog 3; STING—stimulator of interferon genes; TGF-ß—Transforming growth factor-beta; Wnt—Wingless/integrated; ΔNp63—p63 isoform lacking N-terminal domain.

Despite the obvious link between DNA repair pathways and the CSC phenotype, the mechanisms mediating and modulating this crosstalk remain only partially understood. Notably, the double-edged role of the DDR in tumor evolution and therapy resistance suggests a complex relationship that goes beyond the elimination of treatment-induced DNA damage. As the latter topic has already been intensively discussed [11,31,33], this review focuses on the more minor known functions of the DDR in CSC induction and maintenance (Figure 1). This report emphasizes the importance of DNA repair proteins to prevent replication stress and provides various examples for the involvement in CSC-related transcription, signaling, and ROS detoxification. Moreover, we explore the link between the DDR and the antitumor immune response in the context of CSCs and discuss recent developments in targeted therapies.

## 2. Adaptation of CSCs to Replication Stress

Replication stress (RS) is a multifactorial phenomenon with significant implications for the genome stability. Prevention of RS is ensured by a complex surveillance network, the S-phase checkpoint. This network splits into the two sub-pathways detection and signaling of DNA damage (DDR) and the replication checkpoint [28,34,35]. The formation of aberrant replication fork structures leading to single-stranded DNA (ssDNA) triggers the DDR, which is primarily mediated by the central S-phase kinase ataxia telangiectasia-mutated and Rad3-related (ATR). ATR and its downstream effectors, particularly checkpoint kinase 1 (CHK1), stabilize and reactivate stalled replication forks after successful DNA repair to prevent DNA damage and genome instability. Both pathways are highly overlapping, as DNA damage caused by RS delays the progression of replication forks or provokes their collapse. Activation of the Ataxia telangiectasia mutated (ATM) kinase only occurs through collapsed replication forks leading to DNA double-strand breaks (DSBs) [36]. The importance of error-free DNA repair for replication-associated DNA damage is expressed by the presence of de facto all DNA repair pathways at replication forks in CSC (reviewed in [6]). Defects in the DDR genes ATR or ATM alter precancerous cells to CSCs that exhibit increased RS in a Mitogen-activated protein kinase (MEK)/Extracellular signal-related kinase (ERK) signaling-dependent manner in the presence of Cyclin E overexpression [37]. CSCs show that they can tolerate extensive genotoxic stresses, including RS. Consequently, resistance to irradiation and chemotherapeutic agents occurs frequently. How RS is caused and which strategies CSC use to cope with RS is the focus of this chapter.

### 2.1. Endogenous Causes of Replication Stress

Possible endogenous sources of RS include increased reactive oxygen species (ROS) [6,38,39] or availability of nucleotides (dNTP; deoxyribonucleotide triphosphate) [40]. Both lead to activation of the DDR. Other causes include repetitive sequences, such as micro- and minisatellites, and sequences capable of forming non-canonical B-DNA conformations. Most studied are intra-molecular triplex DNA and G-quadruplexes [34]. When replication forks encounter such roadblocks’, replicative helicase and polymerases are blocked and the DDR is activated. The activated DNA repair processes are template switching, homologous recombination (HR), and trans-lesion synthesis, supported by helicases including FANCJ (Fanconi anemia complementation group (FANC) J protein).

Collision of replication and transcription is increasingly considered the major source of RS and occurs when both processes access the same gene sequence simultaneously [41]. RNA-DNA hybrids, known as R-loops, are a widely recognized source of RS and genomic instability. Elimination of R-loop-dependent DNA damage is accomplished by the nucleotide excision repair (NER) DNA repair endonucleases XPF (Xeroderma pigmentosum, complementation group F, also known as FANCQ) and XPG (Xeroderma pigmentosum group G-complementing protein). The topological stress created by the collision of replication and transcription machinery conditions the stabilization of R-loops, leading to replication fork stalling [42]. Cells defective in BRCA2 (Breast cancer type 2 susceptibility protein), FANCD2, or FANCA, exhibit R-loop-dependent DNA breaks [43,44], suggesting that a major function of the FANC pathway is to protect R-loop-containing sites. Moreover, several observations in tissue stem cells and CSC show that this R-loop-induced RS is counteracted by increased expression of the chromatin remodeling complex INO80 [45].

Other causes of RS by transcription-replication interference arise at so-called common fragile sites (CFSs), chromosomal regions attributed to a slowdown of replication. They nest in very large genes. Accordingly, transcription of large genes cognitively often correlates with CFS instability. In addition, CFSs have a low density of replication initiation events and are replicated very late in S-phase. These processes lead to replication slowdown and may be compensated by replacement of chromatin-bound mini chromosome maintenance protein 2 (MCMs), which provokes increased replication initiation events. Additional fragile sites, known as early fragile sites (ERFs), also lead to DNA damage and occur in early S-phase when genes are transcribed in an overlapping manner, confirming the conflict of replication and transcription triggering RS [34].

### 2.2. How CSCs Deal with RS and Consequences for Treatment Resistance

The underlying cause of increased DDR in CSCs was mainly attributed to activation by ROS, which promoted increased PARP1 activity and repair of single-strand breaks [6,38,39]. However, recent studies did not show ROS-mediated activation of the DDR in glioblastoma [46,47]. Here, reduced replication speed, asymmetric bidirectional replication forks, and increased stalling of replication factories led to DDR activation with consequent radioresistance in CSC. This was caused by increased formation of RNA/DNA intermediates through increased expression of “very long genes” (VLG) [47]. Increased replication stress by DNA-RNA intermediates was also observed in breast CSCs. In this study, the observed effect was triggered by up-regulated c-Myc expression. The resulting RS was compensated by increased expression of MCM10 and consequently increased activation of replication origins [48].

Regardless of the causes, whether reduced ROS, RNA/DNA hybrids due to increased transcription, or oncogene expression, the DDR appears to be the key vulnerability of CSCs, favoring the use of DDR inhibitors such as prexasertib for CSC-targeted therapy. Observed resistances could be overcome by combined targeting of RAD51 (Radiation sensitive 51) or MRE11 (Meiotic recombination 11 homolog A) to selectively sensitize primary colorectal CSCs to the CHK1/2 inhibitor prexasertib [49,50]. Observations from embryonic stem cells (ESCs) indicate promising results from combined treatment with ATR and ATM inhibitors. It was also observed that the up-regulated transcription factor MYBL2 (Myb-like protein 2) activates ATM and suppresses replication stress. Consequently, loss of MYBL2 or inhibition of ATM in ESCs leads to replication fork slowing, increased replication fork stalling, and increased origin firing. This finding suggests that in addition to ATR-mediated DDR, a MYBL2-MRN-ATM replication stress response pathway in ESCs could be used to control DNA replication initiation and genome stability [51]. In addition, immunotherapeutic approaches would be promising to pursue, as a direct effect of ATR activity on programmed cell death 1 ligand (PD-L1) expression and stability has been observed since ATR inhibition results in down-regulation of PD-L1 protein levels [52,53].

## 3. The Role of DNA Repair Proteins in the CSC Induction and Maintenance

Despite the emergence of targeted therapies and immunomodulatory approaches, DNA-damaging agents such as ionizing irradiation and platinum-based chemotherapeutics are still a primary strategy for the treatment of various cancers [54,55]. Thus, many studies investigated the expression and activity of DDR factors as determinants of resistance towards these therapies [56,57,58]. Interestingly, an increasing number of reports demonstrate that alterations in DNA repair gene expression equally modulate the cancer cell self-renewal capability, in vivo tumorigenicity, and invasiveness—characteristic properties of CSCs. At first glance, the connection between DNA repair and the cellular stemness program might not be obvious. Yet, accumulating evidence suggests that DNA repair genes possess many regulatory functions in addition to their canonical role in the DNA repair process. This chapter describes selected examples of stemness regulation via co-regulation of transcription, interference with CSC-related signaling, and ROS detoxification and discusses the implications of DNA repair gene expression modulation for the CSC phenotype.

### 3.1. Co-Regulation of Gene Transcription

As with their non-malignant counterparts, the stemness phenotype in CSCs is orchestrated by master transcription factors regulating a complex network of signaling pathways. The enhanced expression of pluripotency-related transcription factors, such as KLF4 and Myc, has been shown to transform differentiated tumor cells into cells with CSC properties [59,60,61].

Thus, the corporation with these transcription factors enables DNA repair proteins to influence the CSC cellular program at the apex of the regulation hierarchy.

Interestingly, several DNA repair proteins have been reported to bind to and potentially stabilize pluripotency-related transcription factors. An example for direct protein-protein interaction is the Telomeric Repeat Binding Factor-2 (TRF2), a component of the telomere-protecting shelterin complex, and the Yamanaka transcription factor Krüppel-like factor 4 (KLF4) [62]. Although the role of TRF2 in maintaining telomere integrity has long been known, its extra-telomeric functions in DNA repair, migration, and CSC maintenance have only recently been investigated [62,63,64]. Although TRF2 inhibits both unwanted non-homologous end-joining (NHEJ) and HR at the telomeric ends, its function on non-telomeric DNA includes only the suppression of NHEJ. In the latter context, TRF2 surprisingly was reported to support HR-dependent repair of DSBs, presumably by facilitating the strand invasion process [63]. Yet, the mechanisms underlying its marked impact on CSC marker expression are not elucidated in detail. Interestingly, in silico analyses indicate that TRF2 can physically interact with KLF4 [62].

KLF4 plays a critical yet highly context-dependent role in the induction and maintenance of CSCs in different tumors, while it exerts tumor suppressor function in others [61,65,66]. For example, in liver carcinoma and osteosarcoma, KLF4 has been linked to transforming non-CSCs into tumor cells with enhanced stem-cell marker expression, and metastatic capacity [61,65]. KLF4 protein levels increase after chemotherapy, potentially mediating therapy-related CSC induction [65]. The protein-protein interaction with TRF2 could provide a missing link between DNA damage and KLF4 induction upon treatment. This hypothesis is supported by previous results showing that in glioblastoma, TRF2 can bind to and increase protein stability of another CSC-related transcription factor, the repressor element 1 silencing transcription factor (REST) [67].

Due to its crucial function in the repression of neuronal differentiation, REST protein stabilization is critical for maintaining pluripotency in glioblastoma CSCs. The binding of TRF2 inhibits the Lys48-polyubiquitination of REST, thus preventing its proteasomal degradation [67].

A strikingly similar mechanism was proposed for the interaction of the HR protein homologous-pairing protein 2 (HOP2, or PSMC3IP, GT198) and the stress-related activating transcription factor 4 (ATF4) [68]. As an integral component of the HR machinery, HOP2 exerts two main functions in the repair of DSBs. In a complex with MND2, HOP2 stimulates the recombinases RAD51 and DNA Meiotic Recombinase 1 (DMC1), while HOP2 alone is presumably involved in the strand invasion process [69]. In contrast, the non-repair-related functions of HOP2 are less well described. For ATF4, an enhanced expression has been associated with therapy resistance and CSC characteristics of pancreatic cancer as well as triple-negative breast cancer (TNBC) [70,71]. Heterodimerization of ATF4 with HOP2 does not increase its DNA binding capacity, but its protein stability, thus indirectly enhancing ATF4-dependent transcription [68]. Interestingly, this interaction is mediated by the respective leucine zipper (bZIP) domains of both proteins. In contrast to bZIP transcription factors such as ATF4, HOP2 itself cannot bind DNA in a sequence-specific way since it lacks the adjacent basic region [68,72].

As the DNA binding capability of repair proteins is generally not restricted to specific target genes, interactions with transcription factors could serve as a mechanism to convey specificity in the regulation of transcription. On the other hand, stabilization of pro-survival and pluripotency-related transcriptional factors by DNA repair proteins could potentially enhance CSC survival and preserve stemness upon DNA damage induction. For example, ATF4 is known to balances anti- and pro-apoptotic gene expression in the response to acute or persistent stress [73]. Interestingly, the switch between both transcriptional programs can partially be attributed to the different ATF4 dimerization partners. Moreover, stabilization by protein-protein interaction is a reported mechanism of posttranslational regulation of the ATF4 protein [73].

Similarly, Nijmegen breakage syndrome protein 1 (NBS)1, an integral component of the MRN complex, stabilizes the hypoxia-inducible factor 1 α (HIF1α) in response to irradiation-induced DNA damage [74]. The MRN complex, composed of the MRE11, Rad50 and the NBS1 proteins, is a key mediator of the ATM- and ATR-related DDR. Functioning in both DNA damage sensing and the actual repair process, the MRN complex crucially regulates initial and long-term DDR towards endogenous and treatment-induced damage [75]. Mutations of the MRN genes have been associated with a wide range of cancers, including non-Hodgkin lymphoma, leukemia, breast, and ovarian cancer [76]. Impaired MRN function promotes tumorigenesis due to continuous genomic instability. On the other hand, a MRN deficiency potentially sensitizes tumor cells to DNA-damaging treatment and causes synthetic lethality in combination with inhibition of the PARP [74]. Importantly, the HIF1α protein level is a subject of intense regulation depending on the oxygenation status, and aberrant stabilization by NBS1 might induce EMT, invasion and other characteristics of the CSC phenotype [74,77].

Moreover, some DNA repair proteins might up-regulate protein levels of CSC-related transcription factors and act as a transcriptional co-activator or co-repressor. For example, the HR protein BRCA1 (Breast cancer 1, early onset) has been shown to direct the transcriptional program of the tumor suppressor p53 towards treatment survival, as described in more detail below [78] (Section 3.3) The canonical function of BRCA1 in DNA repair involves different steps of the HR pathway, e.g., RAD51-dependent strand invasion and D-loop formation, as reviewed in detail elsewhere [23]. Consequently, loss-of-function mutations of BRCA1 are one of the best-investigated genetic alterations that predispose to cancer. In addition to its crucial role in preventing genomic instability, BRCA1 acts as a co-repressor for Myc target genes, which in the absence of BRCA1, promote the aggressive basal-like phenotype in breast cancer [79]. Taken together, several reports suggest that direct interactions of DNA repair proteins and pluripotency-related transcription factors could potentially function as an adaptive mechanism that couples DNA damage to the induction of a pro-survival, CSC-promoting transcriptional program.

Due to the increased availability of complex interactome analysis, recent studies often report various interactions of DNA repair proteins with transcription factors and downstream effectors of CSC-related signaling pathways [80,81]. Yet, the consequences for transcription factor activity and signal transduction remain largely unknown. Thus, treating these interactions not as simple secondary results but as a potential cell-fate regulator could shed additional light on the complex relationship between the DDR and the CSC phenotype.

### 3.2. Regulation of EMT, Survival, and Transformation by Interference with CSC-Related Signaling

It is well known that deficiencies in DNA repair pathways, such as the FANC/BRCA pathway or the DNA mismatch repair (MMR), predispose to cancer due to increased genomic instability. However, the overexpression of DNA repair genes is also often associated with malignant transformation [78,82,83,84,85]. A rapidly growing body of evidence suggests that the altered expression of DNA proteins promotes EMT, metastasis, and de-differentiation of tumor cells by interfering with major CSC-related signaling pathways. Due to the numerous reports of possible interactions, the following paragraphs focus on a few well-investigated examples.

#### 3.2.1. ATM- and ATR-Related Signaling

The DNA damage is sensed by the ATR and ATM serine-threonine protein kinases, which activate the downstream checkpoint kinase proteins CHK1 and CHK2. All of them are highly activated in the different CSC populations. In particular, up-regulation of ATM was shown in breast and cervical cancer [86], HNSCC [87], as well as in glioblastoma [15] CSC populations. It is becoming increasingly evident that ATM has a broader biological function than DDR signaling and contributes to cellular metabolism, chromatin reprogramming, autophagy, and oxidative stress in various normal and cancerous tissues.

In recent studies, activation of DNA double-strand breaks (DSBs) and DDR signaling was shown as an essential part of the oncogene-induced malignant transformation of human fibroblasts [88]. Surprisingly, activation of ATM kinase was a critical component of this transformation as it mediated transcriptional activation of genes involved in stem-cell regulation and carcinogenesis by enabling chromatin access for the oncogenic transcriptional regulators. This study showed that this ATM-dependent transcriptional reprogramming could be attributed to the KRAB-associated protein 1 (KAP1) protein, one of the ATM phosphorylation targets. KAP1 is a part of the protein complex mediating local heterochromatin formation [89] and is phosphorylated by ATM at the Ser824 in response to DNA damage. The ATM-dependent KAP1 phosphorylation drives chromatin relaxation allowing DNA repair in the locus of the DSB formation [90]. In parallel, this phosphorylation enables chromatin accessibility for the oncogenic transcriptional factors driving ATM-mediated cell reprogramming [88]. Furthermore, activation of ATM after radiation-induced DNA damage leads to the consequent activation of transforming growth factor-beta-activated kinase 1 (TAK) and c-Jun N-terminal kinases (JNK) kinases and c-Jun/AP-1-dependent transcriptional activation of PRNP gene encoding for cellular prion protein (PrPC). Activation of this ATM/TAK1/PrPC pathway mediates tumor cell radioresistance and is a known regulator of CSC functions [91].

However, DDR genes such as ATM paradoxically regulate CSC populations independent of the DNA damage. For example, a recent study suggests that CSCs in triple-negative breast cancer (TNBC) rely on the DNA DSB-independent activation of ATM kinase for metabolic reprogramming [92]. This study demonstrated that DNA damage-independent ATM activation in TNBC CSCs induced an energy metabolism reprogramming by up-regulation of the glycolysis-associated genes Glucose transporter type 1 (GLUT1) and Pyruvate kinase PKM2 and the TCA (tricarboxylic acid cycle)-related gene Pyruvate dehydrogenase PDHa through STAT5 (signal transducer and activator of transcription 5)/FOXP3 (forkhead box P3) signaling. This metabolic reprogramming results in increased production of acetyl-CoA and consequently induces epigenetic resetting by the acetylation of specific lysine residues in histone H4. These ATM-induced epigenetic modifications are associated with activating the CSC gene expression, enriching CSC populations, and promoting tumor formation and growth in mouse xenograft models [92]. ATM also mediates phosphorylation and stabilization of Zinc finger e-box binding homeobox 1 (ZEB1) a transcription factor driving EMT, tumor invasion, metastasis, and therapy resistance [93,94]. ZEB1 was shown to increase radioresistance through CHK1 stabilization [94] and suggested as a regulator of breast CSCs [95]. Another study showed that ATM contributes to maintaining breast CSC populations by up-regulation of the autophagy-related genes such as autophagy-related 4C cysteine peptidase (ATG4C). Activation of the ATG4C expression is essential to sustain autophagic flux and mammosphere formation [96].

All in all, these observations indicate that ATM plays a multifaceted role in CSC regulation. Therefore, its targeting can be a promising strategy to eliminate CSC populations and increase the efficacy of conventional therapies.

#### 3.2.2. PI3K/Akt Signaling and DNA Repair Proteins

The Phosphoinositide-3-kinase (PI3K)/Akt signaling pathway is one of the major regulators of cellular processes associated with stemness, EMT, and proliferation. PI3K-dependent phosphorylation of the protein kinase Akt leads to Akt activation and subsequent signal transduction via multiple mediators, such as mTOR (Mammalian target of rapamycin), FOXO (Forkhead box O) transcription factors, and glycogen synthase kinase 3 (GSK3) [97]. GSK3 phosphorylation and inhibition can, in turn, activate WNT (Wingless-type MMTV integration site family)/ß-catenin signaling and Snail-mediated EMT [98,99]. Moreover, the key negative regulator of the PI3K/Akt signaling axis, phosphatase and tensin homolog (PTEN), prevents replication fork progression under stress conditions and therefore prevents genomic instability because of replication stress [100]. Consequently, interference with the PI3K/Akt pathway can induce the CSC phenotype and enhance the metastatic potential of tumor cells [99,101].

In line with the initially described dual role of DNA repair genes in CSC induction, the overexpression of MRN complex components is also associated with transformation and acquired CSC characteristics [84,85]. Importantly, in particular tumor entities such as non-small cell lung cancer, hepatocellular carcinoma, and esophageal cancer, overexpression of NBS1, one of the MRN components, occurs at a much higher frequency than loss-of-function mutations [76,84]. Mechanistically, NBS1 overexpression promotes the CSC phenotype and metastatic potential by up-regulation of PI3K activity [84]. Consequently, AKT is phosphorylated and activated, leading to enhanced Snail expression by inhibition of GSK3. Snail, in turn, regulates multiple EMT genes and induces the matrix metalloproteinase-2 (MMP2), thus supporting metastasis formation [85]. Interestingly, this study showed that not all metastatic HNSCC tumors with enhanced NBS1 levels also express Snail, suggesting that NBS1 overexpression activates additional EMT- and metastasis-related pathways [85]. As described in the Section 3.1, NBS1 can stabilize HIF1α upon DNA-damaging treatment [74]. Moreover, NBS1 has been shown to interact with Notch signaling during neuronal development, although in an inhibiting manner [102]. As with NBS1, high expression of MRE11, another member of the MRN complex, was also associated with Akt activation independently of its function in the MRN complex-mediated DNA repair [82]. Consequently, while the DNA repair function of MRE11 determines its contribution to treatment resistance, it was dispensable for the enhanced proliferation and migration upon MRE11 overexpression.

These results suggest at least partial independence of the functions of MRN complex components in the prevention of genomic instability and the regulation of CSCs signaling. Thus, tumorigenesis related to loss-of-function mutations or protein overexpression might differ in the underlying mechanisms. Pharmaceutical inhibition of NBS1 or other MRN complex components should therefore be investigated by considering the initial expression levels, and genomic integrity should be closely surveyed [82,85]. As a more reasonable treatment strategy for MRN overexpressing tumors, inhibition of PI3K/Akt signaling or other downstream pathways could be considered [84,85]. However, regarding the affected signaling pathways, tumor entity-specific crosstalk should be counted. Although MRE11-dependent transformation in breast cancer was linked to signal transducer and activator of transcription 3 (STAT3) signaling, no such association was found in oral cancer [82,103]. Instead, Akt pathway activation in MRE11 overexpressing oral cancer was mediated by the C-X-C chemokine receptor type 4 (CXCR4), suggesting CXCR4 inhibitors as a potential treatment option specifically for this tumor entity [82].

Taken together, the involvement of the MRN complex components in PI3K/Akt signaling can be considered to be an example of the potential benefits of molecular tumor profiling that goes beyond screening for germline mutations. However, the mechanistic studies on the effect of altered DNA repair protein expression have certain limitations. First, in vitro experiments on cell lines cannot comprehensively reflect the clonal diversity present in a tumor [104]. The occurrence of different subpopulations of bulk tumor cells and CSCs with potentially different MRN expression levels could lead to a less uniform outcome of PI3K/Akt signaling inhibition. Second, short-term down-regulation of DNA repair genes, e.g., by siRNAs, cannot reflect the potentially acquired compensatory mechanisms in tumors harboring inactivating mutations. Third, thinking of loss-of-function mutations only in the context of genomic instability would be an oversimplified view of its diverse consequences. Finally, depletion of a DNA repair protein can also activate CSC signaling.

In contrast to NSB1, loss of function of the key HR protein BRCA1 is associated with continuous activation of the PI3K/Akt signaling axis [105]. In breast cancer, reduced BRCA1 expression or mutation leads to accumulation of phosphorylated Akt (pAkt) [105,106]. In BRCA1 wild-type cells, pAkt levels are controlled by different mechanisms: BRCA1/BARD1(BRCA1 associated RING domain 1)-mediated ubiquitination and proteasomal degradation, stimulation of phosphatase 2A (PP2A) activity, and subsequent de-phosphorylation of pAkt, as well as a potentially altered PI3K activity [105,107,108]. Previous reports describe the BRCA1/BARD1 heterodimer as a complex with ubiquitin E3 ligase activity. As reviewed elsewhere, the ubiquitin ligase activity of BRCA1/BARD1 is not only required for their DNA repair function [23]. Instead, the posttranslational modifications of cell cycle regulators and the estrogen receptor α (ERα), as well as other breast cancer-related proteins, support an additional tumor-suppressive role of BRCA1/BARD1 [23]. For pAkt, polyubiquitination by BRCA1/BARD1 leads to its degradation, thus influencing the pAkt concentration in relation to the active non-phosphorylated Akt [108]. Considering the importance of PI3K/Akt signaling for CSC induction and maintenance in breast cancer, its long-term activation could contribute to developing the treatment-resistant, basal-like phenotype in BRCA1-mutant tumors [99]. Therefore, inhibition of the PI3K/Akt/mTORC signaling axis offers a promising therapeutic approach to target radio- and chemo-resistant CSCs in the context of BRCA1 deficiency [105,106].

#### 3.2.3. TGF-ß/SMAD Signaling and FA/BRCA Proteins

In non-transformed adult stem cells such as hematopoietic stem cells (HSCs), crosstalk between the DDR and pluripotency-related signaling is required to ensure a stable stem-cell population with an intact genome [11]. FA is a rare hereditary disease caused by mutations in genes of the FA/BRCA pathway [25]. The FA/BRCA proteins play a crucial role in the coordination of the DDR and the efficient execution of DNA repair. The canonical function of the FA pathway is the interstrand crosslink repair (ICL). Upon DNA damage, phosphorylation of multiple FA proteins by DDR kinases such as ATR and CHK1 serves as a signal for the assembly of the FA core complex. Next, the FA complex formation leads to the mono-ubiquitination and consequent activation of a heterodimer composed of FANCD2 and FANCI. Mono-ubiquitinated/activated FANCD2-FANCI, in turn, recruits multiple effector proteins of the DDR. The DDR pathways which are directly or indirectly regulated by an activated FA pathway include not only ICL, but also HR, NER, and replication-associated DNA repair [25]. Thus, impairment of the FA pathway leads to substantial genomic instability. FA patients suffer from severe anemia, developmental defects, and a predisposition to leukemia and several solid tumors [25]. Interestingly, FA-associated HNSCC tumors, which are the most frequently diagnosed solid tumors in FA patients, exhibit increased CSC numbers compared to sporadic HNSCCs. [15] Although the continuous genomic instability in adult HSCs crucially contributes to FA bone-marrow failure, recent studies suggest that this effect is augmented by the up-regulation of apoptosis-related pathways [109,110].

An increased expression of the transforming growth factor-beta (TGF-ß) and its downstream effector SMAD3 (Mothers against decapentaplegic homolog 3) was found in FANCD2-mutant hematopoietic stem cells (HSCs) [110]. The authors showed that this hyperactivation of the TGF-ß signaling axis at least in part mediates their sensitivity towards endogenous and exogenous DNA-damaging agents. Consequently, TGF-ß inhibition enhanced HSC survival upon genotoxic stress by promoting HR-mediated DNA repair [110]. Similarly, in breast cancer, TGF-ß signaling was shown to repress multiple DDR genes, including BRCA1, ATM, and MSH2 [111]. Moreover, binding of SMAD3 inhibits the DNA repair function of BRCA1 [112]. Therefore, hyperactivation of the TGF-ß/SMAD signaling axis induces genomic instability in breast cancer cells with wild-type BRCA1 [111,112]. As these cells exhibit a dysfunctional DNA repair similar to BRCA-mutant tumor cells, they are also highly sensitive towards PARP inhibition [111]. In contrast, in malignant glioma, TGF-ß2 promotes radioresistance by transcriptional up-regulation of DNA repair genes [113].

Importantly, FA/BRCA proteins can also modulate SMAD signaling. FANCD2 has been shown to bind to the promoter of the SMAD1 gene, presumably inhibiting its expression [110]. Moreover, BRCA1 and BRCA2 are involved in SMAD-dependent transcription by the binding of SMAD3 and co-activation of its target genes [112,114]. Taken together, these results suggest that crosstalk between TGF-ß/SMAD signaling and the FA/BRCA pathway contributes to cell-fate decisions in HSCs and cancer cells upon genotoxic stress, therefore determining their sensitivity towards DNA-damaging treatment.

#### 3.2.4. p53-Related Signaling and FA/BRCA Proteins

p53 is one of the most investigated tumor suppressor proteins, with fundamental functions in regulating proliferation, metabolism, growth, and apoptosis [115]. In ESC, p53 ensures genomic stability by inducing the differentiation program upon DNA damage, thus eliminating cells with potentially oncogenic alterations from the stem-cell pool [116]. On the other hand, in a limited context, the broad involvement of p53 in proliferation- and stemness-associated pathways might also promote CSC induction and maintenance. For example, in colorectal cancer, p53 mediates the activation of the WNT/β-catenin signaling axis upon Fluorouracil treatment and thus potentially contributes to CSC induction after chemotherapy [117]. As a key player in preventing malignant transformation, the TP53 gene is the most frequently mutated gene in human tumors. In addition to loss-of-function mutations, alterations with a dominant-negative effect and oncogenic gain-of-function mutations are known [115]. As extensively reviewed by Shetzer and colleagues, gain-of-function mutant p53 contributes to the CSC phenotype by promoting various CSC-related processes including EMT, proliferation, apoptosis evasion and drug resistance [118]. Therefore, interactions of DNA repair proteins with p53 signaling have the potential to mediate CSC characteristics in different tumor entities.

In FA patients, p53 contributes to bone-marrow failure and developmental abnormalities via proliferation stop and apoptosis induction in adult hematopoietic stem cells and embryonal tissues [109,116]. The activation of p53 signaling can be considered an anti-transformative effect in FA. Yet, the continuous genomic instability might eventually lead to p53 mutation. Dysfunctional p53, in turn, causes first an improvement in anemia, often followed by the development of leukemia [25,119]. In line with these reports, a heterozygous p53 deficiency facilitates the formation of epithelial tumors in FANCD2 knockout mice [120]. Mutations of the important HR genes BRCA1 and BRCA2 occur not only in FA patients. A heterozygous loss-of-function mutation crucially predisposes to breast and ovarian cancer [23]. Interestingly, p53 mutations are closely linked to BRCA1-associated tumorigenesis, suggesting that abrogation of p53-induced apoptosis is critical for the survival of genetically unstable BRCA1-deficient cells [121].

Several other observations underline the importance of BRCA1 and p53 crosstalk for malignant transformation. As briefly mentioned above, BRCA1 can stabilize p53 and act as a transcriptional co-activator of p53 target genes [78]. In contrast to stabilization by treatment-induced DNA damage, binding of BRCA1 shifts the p53 transcriptional response from apoptosis-related genes towards cell cycle arrest and DNA repair. Therefore, upon DNA damage, overexpression of BRCA1 promotes p53-mediated reversible growth arrest rather than apoptosis induction. This effect presumably contributes to enhanced therapy resistance in BRCA1-overexpressing cells. Thus, the elimination of BRCA1 and simultaneous p53 stabilization by DNA-damaging treatment augmented p53-dependent apoptosis [78]. In contrast, BRCA1 overexpression has also been reported to induce p53-dependent apoptosis by up-regulation of the DNA damage response protein p53-inducible gene 3 (PIG3), suggesting that the outcome of BRCA1-p53 interaction is context-dependent [122].

Moreover, the consequences of this interaction go beyond transcriptional regulation of p53 target genes. Upon persistent DNA damage, the binding of p53 to the C-terminal region of BRCA1 impairs the interaction with BARD1. Consequently, BRCA1 is exported into the cytosol, thus abrogating its DNA repair function [123,124]. This regulatory mechanism is compromised in sporadic breast cancer with wild-type BRCA1 and mutant p53. In this case, non-physiological retention of BRCA1 in the nucleus is associated with enhanced radioresistance [124]. In addition, BRCA1 is required to prevent aberrant p53 signaling as a consequence of p53 mutations. In cooperation with the transcription factor ΔNp63 (p63 isoform lacking N-terminal domain), BRCA1 destabilizes mutant p53 by inhibiting its interaction with HSP90 [125]. On the other hand, gain-of-function mutations in p53 were reported to increase genomic instability by transcriptional down-regulation of BRCA1 [126]. Collectively, these reports show that both overexpression and loss-of-function mutations of FA/BRCA interfere with p53 signaling. Vice versa, the outcome of FA/BRCA expression alterations critically depends on the p53 mutation status.

#### 3.2.5. p63 Signaling and FANCD2/BRCA1

In addition to p53, FANCD2 and BRCA1 have been associated with another member of the p53 family of transcription factors, p63. Although p63 is rarely mutated, deregulation of its expression is associated with CSC induction and tumorigenesis of different cancers, e.g., breast and bladder cancer [127,128].

Importantly, two main p63 isoforms exist that exhibit opposing roles in tumorigenesis: the TA isoform encoding a transactivation domain and the ΔNp63 isoform. Various reports indicate that while TAp63 is essential for proliferation control and apoptosis regulation, ΔNp63 acts as an oncogene [127,128,129,130]. The ΔNp63 isoform is associated with the stem-cell phenotype in the normal mammary epithelium and in breast cancer CSCs [128]. In the latter setting, a high expression of ΔNp63 promotes the aggressive basal-type identity and metastatic properties, e.g., by regulating Wnt, Hedgehog, and Notch signaling [131,132,133]. However, contradictory studies report that loss of ΔNp63 can also lead to malignant transformation and EMT induction in breast cancer, indicating a complex and highly context-dependent role of p63 isoforms [134,135].

In line with a potential anti-transformative effect of balanced ΔNp63 expression, this isoform has been described as a transcriptional target of BRCA1 and an important mediator of BRCA1-dependent tumor suppression [134]. Consequently, down-regulation of BRCA1 and FANCD2 leads to decreased ΔNp63 expression, EMT induction, and de-differentiation of mammary epithelial cells [135]. Moreover, BRCA1 and ΔNp63 contribute to the regulation of stemness and differentiation signaling in breast cancer by joint up-regulation of the Notch ligand Jagged-1 (JAG1) [136].

In accordance with the reports describing ΔNp63 as an oncogene, up-regulation of ΔNp63 by the inactivated/de-ubiquitinated form of FANCD2 promotes bladder cancer proliferation and invasion [127]. As mentioned above, the function of FANCD2 as a DDR regulator and inducer of DNA repair requires its mono-ubiquitination by the FA core complex. Consequently, reversion of the mono-ubiquitination, among other processes, inactivates the FA pathway when DNA repair is completed. [110]. Importantly, the induction of ΔNp63 expression in bladder cancer is due to transcriptional regulation by de-ubiquitinated FANCD2 protein and not an indirect consequence of abrogated FA signaling [127]. Interestingly, upon DNA-damaging treatment, the activated/mono-ubiquitinated form of FANCD2 up-regulates the expression of the tumor suppressor isoform TAp63 in the skin epithelium [130]. The enhanced TAp63 levels in turn promote cellular senescence, therefore suppressing Ras oncogene-driven squamous cell tumorigenesis. Thus, the opposing targets of inactivated and mono-ubiquitinated FANCD2 could reflect a tissue-specific function. However, it can be speculated that FANCD2 might cause a switch in the expression of tumor-suppressing or oncogenic p63 isoforms. In any case, FANCD2 inactivation due to deregulated FA signaling can promote malignant transformation in tumors without FA-related mutations.

#### 3.2.6. WNT Signaling, Intrinsic Apoptotic Pathway, and Mismatch Repair (MMR)

Hereditary MMR deficiency is more frequently associated with colon cancer and endometrial cancer patients [137]. Typically, the germline mutation of an MMR gene is followed by a somatic mutation of the second allele. Consequently, DNA replication errors accumulate and threaten genomic integrity, especially in regions with high repetitive sequences, e.g., microsatellites. MMR deficiency is also found in a fraction of sporadic colon cancer [138].

The question of why MMR-deficient tumors preferentially arise in the colon can be at least partially answered by its regulatory function in the differentiation of colon crypt stem cells and progenitor cells into epithelial cells [139]. The homeostasis of colon stem cells and their differentiated progeny is tightly regulated via the integration of signals from Notch, bone-morphogenic protein (BMP), and WNT/ß-catenin signaling [140]. Although colon stem cells are exposed to increased WNT levels that regulate their proliferation, BMP signaling is blocked to prevent differentiation. In cooperation with WNT, Notch signaling promotes both proliferation and the stemness phenotype and is involved in cell-fate decisions [140]. As with their non-malignant counterparts, colon CSCs can be defined by an increased WNT signaling, while activation of the opposing BMP4 pathway leads to colon CSC differentiation [141,142].

A recent study revealed that MMR deficiency causes hypermethylation of CpG regions in the Dickkopf 1 (DKK1) gene, which is an antagonist of Wnt signaling [139]. Loss of DKK1 function in epithelial cells of the colon crypts (colon epithelial cells, CECs) leads to enhanced WNT/ß-catenin signaling, thus promoting the stemness phenotype and uncontrolled proliferation. Interestingly, because of MMR deficiency, BMP signaling was also up-regulated in the CECs [139]. Therefore, the authors concluded that alterations in the WNT/ß-catenin and BMP signaling in CECs with dysfunctional MMR disturbs the tightly regulated homeostasis of the stem, progenitor, and differentiated cells [139]. These findings suggest that targeting WNT/ß-catenin signaling could at least in part counteract the transformative effect of MMR deficiency.

Moreover, lack of MMR function has been shown to influence the chemo-sensitivity of different tumor cell lines [143]. In addition to safeguarding genomic stability and inhibiting WNT signaling, the MMR pathway is involved in eliminating base adducts produced by alkylating agents [144]. The recognition of O^6^-alkylguanine lesions by the MMR machinery induces mitochondrial apoptosis in a p53-dependent or -independent manner. Thus, MMR inactivation can prevent alkylating agent-induced apoptosis and promote resistance towards certain chemotherapeutic drugs [143,144]. Interestingly, instead of apoptosis induction, *O^6^*-Methylguanine lesions in MMR-deficient cells lead to the formation of interstrand-crosslinks and necrosis [145]. This effect was shown to depend on the activity of the O6-Methylguanin-DNA-Methyltransferase (MGMT). As necrosis and the subsequent massive inflammatory response constitute an unwanted result of the chemotherapeutic intervention, considering the combined MMR/MGMT status could improve treatment choice [145].

In summary, the described interactions of DNA repair proteins with various CSC-related signaling pathways support a scenario in which functional tumor-suppressive crosstalk requires fine-tuned protein expression, precise posttranslational modification, and coordinated sub-cellular localization of all involved factors. Furthermore, overexpression, inactivation as well as loss-of-function or gain-of-function mutations on both sides can potentially disbalance the regulatory network, leading to malignant transformation, de-differentiation, and therapy resistance.

### 3.3. ROS Detoxification and Redox Signaling

Indirect DNA damage resulting from increased levels of ROS is a major cause of the cytotoxicity effect of irradiation and chemotherapy. On the other hand, ROS induction upon treatment has been linked to EMT induction, activation of pro-survival pathways, and enhanced metastasis-related properties of tumor cells [146,147,148]. Even in untreated condition, ROS originate from intrinsic sources, such as the mitochondrial electron-transport chain, the NAD(P)H oxidase (NOX) and the endoplasmic reticulum [149,150]. Although oxidative stress can act as an inducer of the transformation process, disturbance of the redox homeostasis in stem cells has been shown to promote differentiation [151]. Thus, CSCs are in need to control intracellular ROS levels to protect genome integrity and maintain their self-renewal capacity. To this end, CSCs exhibit enhanced antioxidant mechanisms, such as ROS-scavenging molecules [152,153]. Taken together with increased DDR activity, these adaptive mechanisms enable CSCs to survive ROS-inducing therapy. Although it is generally accepted that high ROS levels activate the DDR, the role of DNA repair proteins in ROS detoxification and redox signaling is less explored [154]. This paragraph discusses examples from the base excision repair (BER) and FA/BRCA pathway as well as their potential interplay.

The apurinic/apyrimidinic endonuclease 1/redox effector factor-1 (Ape1/Ref-1 or Apex1) is an integral component of the BER pathway, e.g., in the removal of alkylated or oxidized bases. Its DNA repair function can be attributed to the C-terminal domain, while the N-terminal domain possesses redox activity [150,155]. Due to its redox activity, Apex1 can control mitochondrial ROS levels and prevent apoptosis upon ROS-inducing treatment [156]. Moreover, Apex1 reduces oxidized forms of key transformation-related transcription factors, such as p53, HIF-1α, and nuclear factor kappa-light-chain-enhancer of activated B cells (NF-κB), therefore enhancing their transcriptional activity [150,155]. Thus, up-regulation of Apex1 in response to ROS-inducing treatment mediates cell-fate decisions. Importantly, only wild-type p53, and not its truncated mutants, can be activated by Apex1 [157]. In contrast, wild-type p53 represses Apex1 transcription [158]. Moreover, in p53-deficient cells, Apex1 acts as a transcriptional repressor of the p53 target gene and cell cycle regulator p21 [158,159]. Consequently, the up-regulation of Apex1 in the context of p53 loss mediates proliferation rather than apoptosis.

In line with its dual role in DNA repair and redox signaling, Apex1 is highly expressed by CSCs and cell lines with acquired radioresistance [160,161]. Interestingly, selective inhibition of the Apex1 redox function was sufficient to sensitize colon CSCs to chemotherapy [161]. In addition to the DNA repair capacity, the efficacy of a genotoxic treatment is crucially determined by the intracellular drug concentration. Apex1 was reported to up-regulate transcription of the ATP-binding cassette subfamily G member 2 (ABCG2), therefore promoting drug exclusion from the cell [161]. Moreover, the redox activity of Apex1 mediates tumor growth and migratory properties of pancreatic cancer cells via induction of HIF1α and NF-κB signaling [162]. Taken together, these studies emphasize the broad influence of the multifunctional DNA repair protein Apex1 on the CSC phenotype and therapy resistance. Therefore, targeting Apex1 could simultaneously promote CSC differentiation and increase sensitivity towards ROS induction [161,163]. On the other hand, the dual role of Apex1 creates potential pitfalls for targeted therapies. For example, depletion of Apex1 protein in p53 wild-type tumors could potentially disbalance the p53/p21-dependent tumor suppressor signaling. However, due to the independent location of both functions in different protein domains, selective Apex1 DNA repair or redox inhibitors are available [155].

Interestingly, ROS induction upon Apex1 depletion can be partially reversed by BRCA1 overexpression [164]. BRCA1 was shown to be involved in ROS detoxification and antioxidant gene expression [164,165,166]. In contrast to wild-type BRCA1, the expression of a breast cancer-related mutant leads to increased ROS levels, suggesting a dominant-negative effect [164]. Interestingly, Apex1 overexpression can also partially rescue ROS levels upon silencing of wild-type BRCA1 [164]. Although these results suggest a potentially compensatory function of BRCA1 and Apex1 in the ROS detoxification process, further studies will be required to unravel the underlying mechanisms.

In addition to Apex1, BRCA1 exhibits crosstalk with antioxidant signaling depending on the NF-E2–related factor 2 (NRF2), an important transcription factor for ROS detoxifying enzymes. Upon oxidative stress, BRCA1 regulates NRF2 levels both by up-regulation of mRNA expression and protein stabilization [165,166]. As with the effect of Apex1 targeting, the cancer-associated mutant BRCA1 could not induce the NRF2-dependent antioxidant response [165]. Importantly, Gorrini and colleagues found that NRF2 can alternatively be activated by estrogen. Thus, the authors suggest that estrogen rescues NRF2-dependent antioxidant signaling and prevents ROS-induced apoptosis in the context of BRCA1 loss [165]. The unexpected link between DNA repair proteins, redox function, and estrogen signaling is further supported by the observation that BRCA1 and Apex1 not only possess potentially overlapping functions in ROS detoxification but are also both required for ERα expression and transcription factor activity [167,168].

Interestingly, not only BRCA1 but also other genes of the FA/BRCA pathway have been associated with ROS detoxification. In FA patients, transcription of multiple antioxidant genes is repressed due to increased DNA damage at their promotors, which impairs the recognition by transcription factors [169,170]. In FA wild-type cells, oxidative stress promotes FANCD2 mono-ubiquitination/activation and subsequent complex formation of FA proteins with the chromatin remodeling factor Brahma-related gene-1 (BRG1). The binding of this complex selectively protects antioxidant gene promotors from ROS-induced damage [169]. Moreover, wild-type, but not mutant FANCG can bind and stabilize the mitochondrial peroxidase peroxiredoxin 3 (PRDX3) [171]. PDRX3 plays a critical role in the detoxification of H_2_O_2_ generated in the mitochondria. To interact with PDRX3, FANCG needs to be in the mitochondria, revealing an additional antioxidant function of FA proteins outside the nucleus [171]. Similarly, the inactivation of FANCD2 sensitizes cells towards oxidative stress [172]. Importantly, this effect was shown to occur independently of its role in the replication-associated DNA repair.

The human homologs of *E. coli* AlkB, Histone H2A Dioxygenases (ALKBH) superfamily is a group of alpha-ketoglutarate and Fe(II)-dependent dioxygenases with different functions in DNA repair and epigenetic regulation [173,174]. Several members of the ALKBH family, such as ALKBH2 and ALKBH8, are strongly associated with tumor progression by inducing apoptosis resistance and regulating proinflammatory signaling and EMT [175,176,177]. In line with these findings, a recent study in HNSCC demonstrated the overexpression of seven out of nine human ALKBH proteins in clinical HNSCC samples, namely ALKBH 1-5, ALKBH8 and fat mass and obesity associated protein (FTO) [178]. Interestingly, ALKBH8 has been shown to possess different functions both in the repair of alkylating agent-induced DNA lesions and the regulation of ROS production by NOX-1 (NADPH oxidase homolog-1) [173,175]. As described above, ROS generation can lead to malignant transformation or apoptosis of tumor cells dependent on the cellular context and the extent of induction. In the case of ALKBH8, its activity promotes the production of low ROS amounts by NOX-1, therefore contributing to oncogenic ROS signaling and tumor progression towards an invasive phenotype [175]. Consequently, ALKBH8 silencing impairs NOX-1 mediated ROS generation and downstream signaling, eventually inducing apoptosis [175].

In contrast, silencing of ALKBH7 is associated with decreased ROS levels and the prevention of programmed necrosis [179]. As a result, ALKBH7 depletion enhances resistance of embryonic kidney cells towards alkylating and oxidizing agents [179]. The discrepancy between the effect of ALKBH7 and ALKBH8 down-regulation could be explained by the different cellular contexts of ROS generation. Although ALKBH8-dependent low ROS production takes place in the context of oncogenic anti-apoptotic signaling, high ROS generation is associated with the ALKBH7-mediated programmed necrosis [175,179]. Programmed necrosis is a cell death mechanism occurring upon extensive irreparable DNA damage, caused, for example, by high doses of alkylating agents. During this process, the hyperactivation of PARP leads to depletion of cellular pools of Nicotinamide adenine dinucleotide (NAD+) and Adenosine triphosphate (ATP), mitochondrial dysfunction, and high ROS generation, which further activates downstream degradation events [180]. In this context, ALKBH7 ensures execution of programmed necrosis by inhibiting mitochondrial function and preventing the reconstitution of NAD+ and ATP levels [179]. Consequently, the authors hypothesized that activation of ALKBH7 could provide a possible treatment strategy to sensitize apoptosis-resistant cancer cells towards alkylating agents and ROS-inducing therapy.

Taken together, DNA repair proteins can influence ROS levels and redox signaling on different levels, via. their own intrinsic redox activity, the modulation of mitochondrial function, or indirectly via stabilization of ROS detoxifying enzymes and transcription regulation. Considering that endogenous and exogenous ROS levels are the main source of DNA damage, crosstalk between DNA repair proteins and ROS-related signaling likely plays a vital role in protecting genomic integrity. Perturbations of these tightly regulated interactions harbor the potential to induce genomic instability, treatment resistance, and pro-survival signaling, thus contributing to CSC evolution.

## 4. The Role of DNA Repair Signaling in the Immune Response against Cancer Cells

Besides the impact of the DDR on the maintenance and plasticity of CSCs, direct and indirect effects of the DDR can modulate antitumor immunity. Importantly, damaged DNA and impaired chromosome segregation during mitosis lead to accumulation of genomic DNA in the cytosol. For example, the DDR-induced release of nuclear DNA into the cytoplasm in prostate cancer cells was dependent on the endonuclease methyl methanesulphonate and ultraviolet-sensitive 81 (MUS81), which cleaves DNA upon stalling of replication forks [181]. Although low levels of cytosolic DNA are usually degraded by DNases such as three prime repair exonuclease 1 (TREX1), an accumulation of nuclear DNA in the cytoplasm can stimulate various DNA-sensing pattern recognition receptors (PRRs). For example, the RNA polymerase III recognizes AT-rich dsDNA and activates retinoic acid inducible gene I (RIG-I), which induces type I interferon (IFN) and the NF-κB pathway [182]. Furthermore, cyclic GMP-AMP synthase (cGAS) also detects cytosolic DNA and produces the second messenger cyclic GMP-AMP (cGAMP), resulting in the activation of stimulator of interferon genes (STING) and subsequent type I IFN production [183]. STING phosphorylation by TANK-binding kinase-1 (TBK1) results in the recruitment of interferon response transcription factor (IRF)3, which is involved in the signaling of type I IFN responses. The TBK1-mediated phosphorylation of IRF3 induces its dimerization, nuclear translocation, and transcription of target genes [184]. By promoting the antigen-presenting capacity of dendritic cells and stimulating the cytotoxic activity of CD8+ T cells and natural killer (NK) cells, type I IFNs can essentially contribute to antitumor immunity [185]. As STING is a key factor of the immune response to DNA damage, it has been shown that many tumors down-regulate STING or exhibit mutations, which leads to a dampened inflammatory response [186]. Recently, Suter et al. demonstrated that the inhibition of the cGAS-STING axis is at least partially mediated by interleukin (IL) 6 and downstream JAK2/STAT3 signaling [187]. Interestingly, IL-6 and STAT3 are key players in the generation and maintenance of CSCs, suggesting a mechanistical link [188]. Besides a type I IFN response, the sensing of cytoplasmic DNA via absent in melanoma 2 (AIM2) facilitates inflammasome formation and generation of IL-1β [189,190].

**Figure 2:**
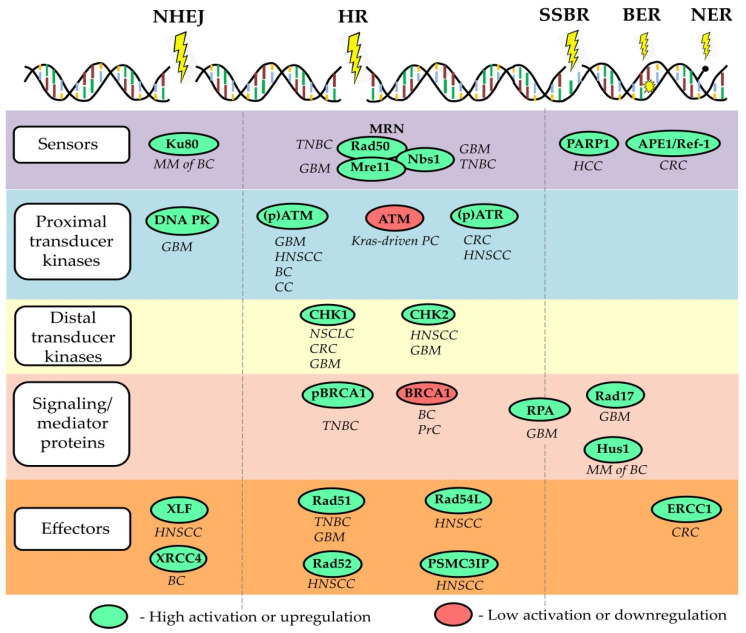
Deregulation of DNA repair proteins in cancer stem cells (CSCs) (examples). Deregulated proteins are involved in the non-homologous DNA end-joining (NHEJ) [87,201,202,203,204], homologous recombination (HR)—mediated DNA repair [15,30,38,86,87,196,197,203,204,205,206,207,208,209] and DNA single-strand break repair (SSBR)/base excision repair (BER) [38,161,201,204,210,211]. DDR proteins are classified as described previously [32,212]. ATM—Ataxia telangiectasia mutated; ATR—Ataxia telangiectasia and Rad3 related; BC—breast cancer; BRCA1—Breast cancer type 1 susceptibility protein; CC—cervical cancer; CHK1—Checkpoint kinase 1; CHK2—Checkpoint kinase 2; CRC—Colorectal cancer; DNA-PK—DNA-Dependent Protein Kinase; ERCC1—Excision repair cross-complementing rodent repair deficiency, complementation group 1; GBM—Glioblastoma; HCC—Hepatocellular carcinoma; HNSCC—Head and neck squamous cell carcinoma; MM—Mouse model; Nbs1—Nijmegen Breakage Syndrome 1 (Nibrin); NER—Nucleotide excision repair; PARP1—Poly(ADP-Ribose) polymerase 1; PC—pancreatic cancer; PrC—prostate cancer; PSMC3IP—PSMC3 interacting protein; RAD5—Radiation sensitive 51; RAD52—Radiation sensitive 52; RAD54L—RAD54-like; Ref-1/APEX1: apurinic/apyrimidinic endodeoxyribonuclease 1; RPA—Replication protein A; TNBC—Triple-negative breast cancer; XLF—XRCC4-like factor; XRCC4—X-ray repair cross-complementing 4.

In addition to cytosolic DNA stimulating classical PRRs, certain molecules involved in the DDR closely interact with the immune system. For example, the DNA repair protein DNA-dependent protein kinase (DNA-PK) prolongs the half-life of the activated form of the transcription factor IRF3, which is involved in the signaling of type I IFN responses [184,191]. Furthermore, DNA-damaging agents induce an ATM-dependent small ubiquitin-like modifier (SUMO) modification of NF-κB essential modulator (NEMO), which fosters IκB kinase complex (IKK) and subsequent NF-κB activation [192]. Although a temporary inflammatory response may promote tumor elimination by activation and recruitment of immune cells, chronic inflammation can in turn lead to further DNA damage, for example by phagocyte-derived ROS, and thus foster tumor progression [193]. Importantly, the DDR also prevents the accumulation of additional cytosolic DNA and the subsequent triggering of cytosolic DNA sensors. As shown by Wolf et al., the DDR proteins RAD51 and replication protein A (RPA) prevent short ssDNA from passing the nuclear membrane and a knockdown of both molecules resulted in a cGAS-dependent activation of the type I IFN response [194]. Depletion of RAD51 is associated with accumulation of cytosolic DNA attributed to excessive degradation of the reverse replication fork by MRE11 exonuclease and consequent activation of STING signaling [195]. Interestingly, RAD51 was shown to be up-regulated in CSCs and drive the resistance of CSCs towards PARP1 inhibitors in TNBC and glioma [196,197], Figure 2. Similarly, the loss of BRCA2 leads to cytosolic DNA accumulation and subsequent cGAS/STING-dependent up-regulation of IFN-stimulating genes [198]. Another study demonstrated that radiation delivered to tumor cells at a high dose in a single fraction induces expression of TREX1 DNA exonuclease leading to the accumulation of cytosolic DNA and activation of cGAS/STING signaling, illustrating the mechanism of radiation-induced innate immune signaling [199]. Furthermore, DNA damage in the S-phase of the cell cycle highly activates STING/TBK1/IRF3 signaling activation and expression of PD-L1 in breast cancer cells that lack functional FA/BRCA-signaling S-phase specific DNA repair mechanism [200].

Besides driving proinflammatory immune responses via STING and NF-κB, the DDR promotes tumor cells to express ligands for the activating NK cell receptors NKG2D (Killer cell lectin-like receptor subfamily K, member 1) and DNAM-1 (DNAX Accessory Molecule-1) via ATM and ATR signaling [213,214]. Functional experiments revealed that the aphidicolin-induced up-regulation of NKG2D ligands on T cell blasts increased their sensitivity towards IL-2-activated NK cells, which was partially reduced by addition of an anti-NKG2D antibody. These results are supported by Soriani et al., who showed that the degranulation of NK cells in the presence of myeloma cells was promoted upon treatment with doxorubicin or melphalan, while the addition of NKG2D and DNAM-1 blocking antibodies reduced the observed effect. Furthermore, a persistent DDR facilitates the senescence of tumor cells accompanied by the secretion of IL-6 and IL-8, which is also known as the senescence-associated secretory phenotype (SASP) [215]. Accumulating evidence suggests that the SASP promotes invasiveness, regenerative capacity and other characteristics associated with the generation of CSCs [216]. It has also been shown that DSBs induce PD-L1 expression by cancer cells via the ATM/ATR/CHK1 signaling axis and the IRF1 pathway [52,53,217]. Therefore, DDR-associated molecules emerged as potential biomarkers predicting the efficacy of checkpoint inhibitor (CPI) treatment. In a cohort of CPI-treated metastatic urothelial carcinoma, the activation of the DDR pathway was associated with a reduced TGF-β signaling as well as an increase in neoantigen load, activated immune cells, and survival time [218]. Another study found that mutations of the DDR pathway were associated with improved overall survival in a colorectal CPI-treated colorectal cancer (CRC) patient cohort, while there was no such correlation in the non-CPI cohort [219].

In conclusion, the DDR can modulate antitumor immunity by facilitating the production of proinflammatory cytokines and promoting tumor cells to express ligands for stimulating NK cell receptors. However, a functional DDR also prevents the accumulation of cytosolic DNA and subsequent activation of the immune system, which suggests particular implications for CSCs, as they frequently overexpress proteins involved in the DDR. Nevertheless, the effect of an altered DDR on the immune response towards CSCs remains to be discovered, as CSCs additionally create an immunosuppressive environment and exhibit various mechanisms to effectively evade the recognition and elimination by the immune system [220].

## 5. DNA Repair in Cancer Stem Cells as a Therapeutic Target

The fundamental properties of CSCs, such as unlimited self-renewal potential, differentiation capacity, and, consequently, tumor-maintaining properties, make them an ultimate therapeutic target for permanent tumor control. Thus, the utmost clinical importance of CSC is proven using CSC-related signatures as reliable prognostic biomarkers and by clinical trials aiming at targeting CSCs in different tumor entities. Targeting DNA repair in CSCs can be suggested as a promising anti-cancer therapeutic strategy as many preclinical studies revealed a high DNA repair capacity in CSCs. Indeed, CSCs in the different tumor entities are shown to be more proficient in the activation of the DDR signaling pathway due to the activation of its various components, including signal sensors, transducers, and effectors, as summarized in a recent review of Schultz et al. and shown in Figure 2. On the other hand, defective DNA repair leads to genetic instability and cancer susceptibility [221]. Here, we will discuss the role of several DNA repair proteins as the most promising emerging targets to eliminate CSC populations and discuss the potential challenges of their clinical translation.

### 5.1. Targeting of ATM and ATR Signaling for CSC Eradication

As discussed earlier, ATM plays multiple biological roles beyond DDR signaling in regulating CSC survival and therapy resistance. Therefore, it can be a promising target to eradicate CSCs and increase tumor sensitivity to conventional therapies. Indeed, several studies demonstrated that chemical inhibition of ATM kinase resulted either in CSC differentiation as in myeloid leukemia [222] or in the CSC sensitization to the conventional treatment such as radiotherapy as in glioblastoma [15,223,224] (Table 1). Several ongoing early-stage clinical trials are currently assessing the safety, tolerability, and preliminary efficacy of the pharmacological ATM inhibition combined with chemotherapy or radiation therapy in patients with solid tumors (Table 1).

Less is known about the role of ATR protein in the regulation of CSCs. Many CSC populations are maintained in vivo in a slow-proliferating or quiescent state, making them a challenging target for the conventional chemotherapeutic drugs that are effective for highly proliferative tumor cells [231]. ATR inhibition with VE-821 drug sensitized quiescent osteosarcoma cells to cisplatin treatment [232]. HNSCC stem cells also showed preferential activation of ATR with or without irradiation [87]. However, similar to ATM kinase, ATR inhibition substantially increased mutagenesis in cisplatin-treated non-cancerous and cancer cells [232]. Inhibition of ATR by caffeine treatment, ATR inactivating mutation, or genetic knockdown resulted in the depletion of CD133^+^ colon cancer stem cells and reduced in vivo tumorigenicity in the xenograft mouse model. Induction of stalled replication forks using DNA interstrand cross-linking agents such as cisplatin and ATR/CHK1 inhibitors such as caffeine and SB218078 synergistically inhibited CD133^+^ CSC population in colon cancer cell lines [228].

The ATR- and ATM-regulated CHK1 and CHK2 kinases also play a role in regulating CSC populations. In particular, radioresistant CD133^+^ glioma stem cells have increased basal activation of CHK1 and CHK2 compared to non-stem-cell cultures [20,46,47]. Rapid and increased activation of CHK1 plays a pivotal role in the chemoresistance of non-small cell lung cancer (NSCLC) stem cells. NSCLC stem cells possess a higher basal level of CHK1 activation and CHK1 phosphorylation in response to chemotherapy such as gemcitabine compared to their differentiated counterparts. A combination of CHK1 inhibitors such as SB218078 or AZD7762 potentiates the effect of conventional chemotherapy, including gemcitabine and cisplatin on NSCLC stem cells in vitro and in xenograft models [229]. In another study, from the same group, a chemical library screening identified the CHK1/CHK2 inhibitor LY2606368 as one of the drugs specifically targeting colorectal CSCs in replication stressed, p53-deficient, and hyperdiploid patient-derived CRC cell cultures in vitro and in mouse xenografts. The inhibitory effect of LY2606368 on CSCs was confirmed independently on RAS-mutated status. The sensitivity of CSCs to LY2606368 depends on the level of the endogenous DNA damage, and experimental p53 knockdown, inhibition of ATM activity, cellular ploidy, or induction of the replication stress by aphidicolin, hydroxyurea, or thymidine sensitized CSCs to the LY2606368 treatment [226].

Treatment of CRC cells with a neo-synthetic bis(indolyl)thiazole alkaloid analog, nortopsentin 234 (NORA234), resulted in an initial inhibitory antitumor effect. However, long-term exposure to NORA234 led to acquired therapy resistance, enrichment in CD44v6-expressing CSC populations, and up-regulation of the DDR proteins, including CHK1 expression. The CHK1 inhibitor LY2603618 has been shown to induce synthetic lethal antitumor effect in combination with NORA234 treatment and eradicated therapy-resistant CSC populations [227].

A growing body of evidence suggests that the DDR depends on nutrient use and intracellular metabolism [233,234]. The combination of the antidiabetic drug metformin and the antibiotic salinomycin inhibited the spherogenic properties of NSCLC cell lines. The effect of this combination therapy was associated with the down-regulation of several oncogenes, including CHK2 expression [230].

Despite several ATR, CHK1, and CHK2 inhibitors entered early-stage clinical evaluation, none of them have been approved for clinical use or even reached late phase clinical trials (Table 1) [235]. Although some of these inhibitors showed acceptable safety and tolerability, their combination with conventional treatment is often associated with increased normal tissue toxicity [236]. On the other hand, combination of Chk1 and ATR inhibition with other DDR and signal transduction inhibitors such as drugs targeting WEE1, PARP1, or broad kinase inhibitors could potentially be better tolerated and more efficient anti-cancer treatment strategies [237,238,239,240,241,242].

### 5.2. Mutation of DNA Repair-Related Genes as a Sweet Point for CSC Targeting

Many components of the DNA repair mediated by HR/FANC involved in maintaining genomic integrity are frequently mutated in different types of cancers. For example, the tumor suppressor gene BRCA1 is often mutated in breast and ovarian cancers. BRCA is a pleiotropic protein with multiple biological functions. Beyond its critical role in DNA repair, BRCA1 regulates gene transcription, cell cycle progression, autophagy, oxidative stress, and cell differentiation [23]. Recent studies demonstrated that BRCA1 is an integrative part of the mammary stem-cell differentiation, and BRCA1 mutations lead to the accumulations of R-loops, the DNA-RNA hybrids structures inhibiting the expression of genes involved in the differentiation of normal luminal progenitor cells. As R-loops are important contributors to genomic instability, these studies demonstrated the role of BRCA1 in the CSC development and breast tumor initiation [243,244]. Indeed, BRCA proteins are involved in regulating CSC populations in multiple tumor entities, as reviewed elsewhere [23]. In breast and prostate cancer cells, knockdown of BRCA1 enhanced CSC characteristics [205,206]. The inhibitory role of BRCA1 for CSCs can be partially attributed to its interaction with epigenetic regulators zeste homolog 2 (EZH2) and histone deacetylase (HDACs) [205,206]. Epigenetic reprogramming with HDAC and EZH2 inhibitors is a promising strategy to eradicate CSCs and sensitize tumors to conventional treatments [245,246,247]. Due to a tight interconnection between BRCA1 and epigenetic mechanisms, BRCA1-deficient breast cancer cells are less sensitive to epigenetic inhibition, such as treatment with the HDAC inhibitor SAHA [205]. At the same time, treatment of prostate cancer cells with the EZH2 inhibitor DZNep abrogated BRCA1-dependent regulation of CSC phenotypes [206]. Furthermore, due to the synthetic lethal interaction between alternative a-NHEJ and HR-driven DNA repair mechanisms, inhibition of the a-NHEJ component PAPR1 induces accumulation of the unrepaired DNA and cell death in BRCA1 mutated tumors deficient for HR-dependent DNA repair [248].

Inactivation of the p53 tumor suppressor is a hallmark of most types of cancer and one of the survival mechanisms for CSC with DNA repair deficiency [30,249]. Under DNA-damaging treatments, activation of p53 is required either for the cell cycle arrest to repair DNA lesions or for the induction of cell senescence and death if DNA damage is unrepairable. In the absence of p53-mediated G1- and G2/M-phase cell cycle arrest, cancer cells harboring DNA lesions are no longer arrested at the G1/S transition and progress through S-phase into the G2 phase and mitosis [250]. Thus, p53 plays a vital role in maintaining genome integrity, and its dysfunction leads to the accumulation of DNA mutations and genome instability. In response to UV treatment, impaired p53 activation in esophageal CSCs was associated with an attenuated G1 and G2/M-phase cell cycle arrest. Thus, it can be a potential mechanism of CSC survival upon DNA toxic therapy and cancer progression [30]. Furthermore, developing p53-activating therapeutic strategies including MDM2 and MDMX inhibition, induction of p53 transcription program by chemical compounds such as CP-31398 and PRIMA-1met, and synthetic lethality caused by p53 loss in combination with inhibition of protein kinases such as WEE1, FYN, and AURKA open further directions for targeting of CSCs deficient for p53 activation [251].

### 5.3. Current Controversy Regarding the Role of DNA Repair Genes as Tumor Regulators and Defenders of Genome Integrity

Enhanced DNA repair and extended cell cycle arrest upon DNA toxic treatments are not always a common feature of CSCs possessing relative radioresistance compared to their non-CSC counterparts [22,30]. Furthermore, up-regulation of a single or several DNA repair genes does not always correlate with more efficient DNA repair mechanisms. For example, a study based on the analysis of The Cancer Genome Atlas (TCGA) database showed that increased expression levels of a gene set, including 23 DNA repair genes, are associated with a deficient HR and inhibition of DNA repair machinery and therefore are indicative for the improved patients’ response to the DNA toxic therapies [252]. Another finding demonstrated that high expression of four DNA repair proteins such as Rif1, PARP-1 Binding Protein (PARI), RAD51, and Ku80 indicates low HR-dependent DNA repair efficiency and is associated with genomic instability and high sensitivity to platinum-based chemotherapy in NSCLC patients [253].

These studies are supported by a recent finding showing that high expression levels of single genes involved in HR-dependent DNA repairs such as POU5F1, PSMC3IP, and RAD54L are associated with better disease-free survival in patients with HNSCC. Furthermore, this study also showed that the transcription factor POU5F1/Oct4 is a marker of HNSCC stem cells and acts as a transcriptional regulator of PSMC3IP and RAD54L expression, suggesting a mechanism of direct regulation of DNA repair by CSC-specific transcriptional factors [208]. A similar observation was also made for DNA repair genes representing the chromosomal (CIN) instability score in patients with HNSCC, indicating that high expression of individual DNA genes did not lead to the therapy resistance [254].

Although several studies showed that ATM inhibition resulted in CSC eradication as discussed before, loss of ATM function serves as a double-edged sword as it might promote malignant transformation and accelerate tumor growth. In particular, a study using genetically modified mouse models showed that mice developing Kras-driven pancreatic cancer showed more aggressive tumor growth in the case of conditional deletion of ATM [255]. Loss of ATM was also associated with enrichment of CSC populations and higher metastatic burden in this mouse model. Consistent with the results obtained from the mouse model, low expression of ATM was correlated with shortened survival in pancreatic ductal adenocarcinoma (PDAC) patients [255]. Another study showed that NSCLC CSCs exhibited diminished DNA-PK and ATM phosphorylation, impaired G2/M and S-phase cell cycle arrest, and decreased DDR and apoptotic response after X-ray irradiation. ATM inhibition in the bulk NSCLC cell cultures decreased cisplatin-induced PARP cleavage [22]. Finally, some DNA repair genes that are highly expressed in therapy-resistant CSCs drive genomic instability. In particular, a recent study suggested that DNA-PK, an essential regulator of NHEJ, is up-regulated in radioresistant glioma CSCs. At the same time, DNA-PK increased radiation-induced genomic instability in CSCs, making them a driving force of tumor evolution and clinical progression [202].

## 6. Conclusions

Tumor stem cells have successfully evolved strategies to adapt to the permanent effects of replication stress, including DNA lesions, unusual secondary DNA structures, limited nucleotide amounts and transcription defects. The central role in this context is provided by the S-phase checkpoint, which controls and coordinates both the cell cycle and DNA repair processes and offers promise as a target for successful tumor therapy, particularly inhibitors of the immune checkpoint.

An increasing number of studies showed that DNA repair genes interact with tumor stemness- and resistance-associated processes on multiple levels, via. co-regulation of transcription, interference with CSC-related signaling and ROS detoxification. Thus, modulation of the expression or activity of DNA repair genes, e.g., by loss-of-function mutations or DDR-activating treatment, potentially has an essential impact on the CSC phenotype. Taking into consideration these non-canonical functions of DNA repair genes could profoundly improve our understanding of the complex relationship between DNA damage and the induction and survival of CSCs.

Besides the role of the DDR in the CSC maintenance and plasticity, it can promote antitumor immune response by mediating the production of proinflammatory cytokines and expression of ligands for stimulating NK cell receptors by tumor cells. It also prevents the accumulation of cytosolic DNA and subsequent triggering of PRRs. Importantly, a persistent inflammatory state can, in turn, contribute to further DNA damage and thus drive tumorigenesis.

All in all, the role of DNA repair proteins in the regulation of CSC therapy resistance and tumor progression is dependent on the level of protein expression, genetic background, and tumor type. Thus, the disparity in the role of single DNA repair genes in the regulation of cancer progression and therapy resistance could pose a challenge to the clinical translation of its chemical inhibitors. Furthermore, the clinical targeting of CSC is challenged by their plastic and reversible nature and the similarity between the molecular features attributed to normal stem cells and CSCs. Therefore, a better understanding of the diverse biological roles of DNA repair-related proteins is needed to develop more efficient cancer treatment, identify more specific prognostic biomarkers, and improve the clinical outcomes of patients with cancer diseases.

## Figures and Tables

**Table 1:** Chemical inhibitors targeting CSC populations or sensitizing CSCs to anti-cancer therapies.

Tumor Type	Chemical Inhibitor	CSC Enrichment and Analysis	Biological Effect	Tumor Model	Clinical Trial (Selected)	Refs
GBM	ATM inhibitor KU55933	Stem-cell-enriching conditions; -Sphere formation	Inhibition of DNA DSB repair after RT; CSC sensitization to RT In Vitro	Patient-derived cell cultures	-NCT03423628: Study to test ATM inhibitor, AZD1390 in combination with radiation therapy for the treatment of brain tumors, Phase 1 -NCT04550104: Study to determine the recommended dose and safety profiles of different DDRis when given in combination with curative intent RT in patients with stage IIB/IIIA/IIIB NSCLC, Phase 1 -NCT02588105: Study will determine the best dose and safety of AZD0156 when given alone or in combination with other agents e.g., Olaparib, irinotecan, Fluorouracil, Folinic Acid in patients with locally advanced/metastatic tumors, Phase 1 -NCT03225105: Study to evaluate the safety, tolerability, PK, PD, antitumor activity of ATM inhibitor M3541 in combination with fractionated palliative RT in patients with solid tumors with malignant lesions e.g., in the thorax, abdominal cavity, head and neck region, Phase 1	[15]
GBM	ATM inhibitor KU60019 KU55933	Stem-cell-enriching conditions	Sensitization of tumor cells to radiotherapy In Vitro; -Down-regulation of Prom1, Bmi1, CD15, Msi1, Msi2, Nanog, Nestin, Oct4 and Sox2 in response to combination of KU60019 and RT	Established cell lines, in vitro sphere formation; Orthotopic mouse tumor models		[223,224]
Mouse model of GBM	ATM inhibitor KU60019 DNA-PK inhibitor NU7441	IHC analysis of CD133+ cells	Sensitization of CSCs to RT in vivo in GEMM	GEMM: primary GBM in Ntv-a;Ink4a–/–;Ptenfl/fl;LSL-Luc donor mice induced by RCAS-mediated somatic gene transfer		[202]
Mouse model of AML	ATM inhibitor KU55933	-Cell morphology	Inducing differentiation of leukemic blast cells	The frequency of blasts and differentiated cells was identified in vitro by morphology		[222]
GBM	CHK1/CHK2 inhibitor DBH	CD133 expression	Sensitization of CSCs to RT In Vitro and in mouse xenograft model	Patient-derived cell cultures; Orthotopic mouse tumor model	n.a.	[38]
GBM	RAD51 inhibitor RI-1	Stem-cell-enriching conditions	Combination of RI-1 and RT increased apoptosis and delayed G2 arrest in radioresistant CSC cultures	Patient-derived cell cultures	n.a.	[225]
GBM	ATR inhibitor VE821 in combination with PARP1/ PARP2 inhibitor Olaparib	Stem-cell-enriching conditions	Inhibition of DNA DSB repair after RT; Inhibition of sphere formation after RT; CSC sensitization to RT In Vitro	Patient-derived cell cultures; In Vitro sphere formation	-NCT03967938: Study to evaluate efficacy of Olaparib in advanced cancers in patients with germline mutations or somatic tumor mutations in homologous recombination genes (1-2018 BSMO), phase 2 -NCT03742895: Study to assess efficacy and safety of Olaparib in patients with previously treated, HR repair mutation (HRm) or HR deficiency (HRD) positive advanced cancers (MK-7339-002/LYNK-002), phase 2 -NCT03212742: Study to investigate the toxicity and efficacy of olaparib and TMZ concomitantly with radiotherapy in first line treatment of unresectable high risk HGG (OLA-TMZ-RTE-01), phase ½ -NCT03167619: A randomized multicenter study to explore the efficacy of olaparib or olaparib in combination with durvalumab in platinum-treated TNBC, Phase 2	[47]
GBM	-CHK1 inhibitor SCH900776 (SCH); -ATM inhibitor KU55933 in combination with PARP1/ PARP2 inhibitor Olaparib -ATM inhibitor VE821 in combination with PARP1/ PARP2 inhibitor Olaparib	Stem-cell-enriching conditions	Inhibition of DNA DSB repair after or G2/M checkpoint activation after RT; CSC sensitization to RT In Vitro	Patient-derived cell cultures; In Vitro sphere formation		[46]
GBM	PARP1/ PARP2 inhibitor Olaparib	In Vitro limiting dilution assays In Vitro limiting dilution assays	Sensitization of tumor cells to RT In Vitro in In Vivo; Inhibition CSC self-renewal In Vitro in In Vivo; Inhibition CSC viability in vitro and tumor growth In Vivo	Patient-derived cell cultures; S.c. xenograft mouse model		[39]
TNBC	PARP1/ PARP2 inhibitor Olaparib	ALDEFLUOR assay	Sensitization to PARP inhibition mediated by RAD51 KD	Established *BRCA1*-mutant cell lines with or without RAD51 KD		[196]
CRC	CHK1 inhibitor LY2606368	Sphere formation	Inhibition of tumor growth In Vitro and In Vivo	Patient-derived cell cultures Mouse xenograft models	-NCT02203513: Single arm study in BRCA1/2 mutation associated BC or OC, TNBC, high grade serous OC, and mCRPC, phase 2; -NCT04032080: Study to evaluate efficacy of LY3023414 and prexasertib in patients with metastatic TNBC, phase 2; -NCT02555644: Combination with chemotherapy and radiation in participants with HNSCC, phase 1	[226]
CRC	CHK1 inhibitor LY2603618 (Rebusertib)	Sphere formation	Apoptosis induction (Annexin V positivity); -Inhibition of tumor cell growth In Vitro	Patient-derived cell cultures with or without *RAD51* KD	-NCT00988858: Study to evaluate efficacy and safety of LY2603618 in combination with pemetrexed in participants with advanced or metastatic NSCLC, phase 2; -NCT00839332: A study to evaluate the dose and efficacy of LY2603618 in combination with Gemcitabine for participants with PC, phase 1/2	[227]
CRC	CHK1 inhibitor SB218078	CD133 expression	Inhibition of CD133+ cell population; Sensitization of CSCs to cisplatin In Vitro	Established cell lines	n.a.	[228]
NSCLC	CHK1 inhibitor SB218078 AZD7762	Sphere formation	Inhibition of tumor growth In Vitro and In Vivo in combination with conventional chemotherapeutic drugs	Patient-derived cell cultures; Mouse xenograft models	-NCT00413686: Study to assess safety of AZD7762 administered alone and in combination with gemcitabine in patients with advanced solid malignancies, phase 1	[229]
NSCLC	Combination of antidiabetic drug metformin and antibiotic salinomycin	Sphere formation	Inhibition of CHK2 (T68) phosphorylation; Inhibition of sphere formation	Established cell lines, in vitro sphere formation	-NCT01579812: Targeting CSC for prevention of relapse in gynecologic patients, phase 2 -NCT02437656: Combination of metformin to neoadjuvant radiochemotherapy in locally advanced RC (METCAP), phase 2 -NCT04387630: Neoadjuvant chemotherapy with or without metformin in early BC, phase 2/3	[230]

Acronyms: AML—acute myeloid leukemia; BC—breast cancer; CHK1—checkpoint kinase 1; CHK2—checkpoint kinase 2; CRC—colorectal cancer; GBM—glioblastoma; CSC—cancer stem cells; DBH—debromohymenialdisine; mCRPC—metastatic castrate-resistant prostate cancer; DDRis—DNA damage repair inhibitors; GEMM—genetically engineered mouse model; HGG—high grade gliomas; HNSCC—head and neck squamous cell cancer; HR—homologous recombination; IHC—immunohistochemical analysis; KD—knockdown; NSCLC—non-small cell lung cancer; OC—ovarian cancer; PC—pancreatic cancer; PD—pharmacodynamic; PK—pharmacokinetic; RC—rectal cancer; RCAS—Replication-competent ALV splice acceptor; RT—radiotherapy; S.c.—subcutaneous; TMZ—Temozolomide; TNBC—triple-negative breast cancer.
